# Post-Stroke Social Isolation Reduces Cell Proliferation in the Dentate Gyrus and Alters miRNA Profiles in the Aged Female Mice Brain

**DOI:** 10.3390/ijms22010099

**Published:** 2020-12-24

**Authors:** Aleah Holmes, Yan Xu, Juneyoung Lee, Michael E. Maniskas, Liang Zhu, Louise D. McCullough, Venugopal Reddy Venna

**Affiliations:** 1 Department of Neurology, McGovern Medical School, The University of Texas Health Science Center at Houston, Houston, TX 77030, USA; aleah.holmes@uth.tmc.edu (A.H.); yan.xu.1@uth.tmc.edu (Y.X.); juneyoung.lee@uth.tmc.edu (J.L.); michael.maniskas@uth.tmc.edu (M.E.M.); Louise.D.McCullough@uth.tmc.edu (L.D.M.); 2 Biostatistics and Epidemiology Research Design Core, Center for Clinical and Translational Sciences, The University of Texas Health Science Center at Houston, Houston, TX 77030, USA; liang.zhu@uth.tmc.edu

**Keywords:** ischemic stroke, social isolation, miRNA, neurogenesis, aging

## Abstract

Social isolation and loneliness are risk factors for stroke. Elderly women are more likely to be isolated. Census data shows that in homeowners over the age of 65, women are much more likely to live alone. However, the underlying mechanisms of the detrimental effects of isolation have not been well studied in older females. In this study, we hypothesized that isolation impairs post-stroke recovery in aged female mice, leading to dysregulated microRNAs (miRNAs) in the brain, including those previously shown to be involved in response to social isolation (SI). Aged C57BL/6 female mice were subjected to a 60-min middle cerebral artery occlusion and were randomly assigned to either single housing (SI) or continued pair housing (PH) immediately after stroke for 15 days. SI immediately after stroke led to significantly more brain tissue loss after stroke and higher mortality. Furthermore, SI significantly delayed motor and sensory recovery and worsened cognitive function, compared to PH. A decrease in cell proliferation was seen in the dentate gyrus of SI mice assessed by bromodeoxyuridine (BrdU) labeling. miRNAome data analysis revealed changes in several miRNAs in the brain, such as miR-297a-3p and miR-200c-3p, which are known to regulate pathways involved in cell proliferation. In conclusion, our data suggest that SI can lead to a poor post-stroke recovery in aged females and dysregulation of miRNAs and reduced hippocampal cell proliferation.

## 1. Introduction

Stroke is the fifth leading cause of death and the primary cause of adult disability in the United States [1]. Worldwide, stroke is responsible for approximately 5.5 million deaths (9% of total deaths) annually [1]. As life expectancy increases, the incidence of stroke will continue to rise [2]. Social isolation (SI) is an independent risk factor for increased all-cause mortality [3,4,5,6,7]. Data from the United States (US) Census shows an increase in the number of people living alone from 17% of households in 1970 to 27% in 2012, and this trend is likely to increase, especially in elderly women [8,9]. Widowed or single older men and women are a demographic group susceptible to neurological disorders [10]. They represent more than 30% of men and 56% of women aged 65 years or older living in the US [9]. SI has been associated with both a higher incidence, accelerated disease progression, and impaired recovery in many vascular and neurological diseases, including stroke [11,12,13,14]. However, the mechanisms mediating the detrimental effects of SI remain largely unknown.

It is now well documented that microRNAs (miRNAs) play an important role in stroke recovery. Interestingly, recent studies have found that miRNAs also mediate many aspects of social interaction [15,16,17]. miRNAs are small non-coding RNAs with 18–24 nucleotides in length that regulate gene expression by directly degrading mRNA or suppressing post-transcriptional protein translation by binding to the 3′-untranslated region of targeted mRNAs [18]. miRNAs are involved in crucial biological processes, including cell differentiation, apoptosis, proliferation, metastasis and metabolism [18,19]. miRNA dysregulation is involved in a wide range of diseases, including obesity, cancer and neurological disorders [20,21,22,23]. Social environments can directly influence miRNA expression, which then triggers a plethora of downstream gene changes [24,25,26]. Manipulation of miRNA expression has been used to influence ischemic stroke recovery in animal models [27,28,29], leading to an increase in neurogenesis. Our previous research supports that a period of post-stroke SI can lead to changes in neurogenesis during stroke recovery [14], which may be due to changes in the regulation of certain miRNAs that target brain-derived neurotrophic factor (BDNF) and other neurotrophic factors. This led us to hypothesize miRNA regulation as a potential mechanism involved in the detrimental effects of SI following stroke.

In this study, we investigated if SI immediately after stroke contributes to changes in ischemic injury and mortality rates in aged females. Moreover, we investigated the effect of SI on functional and cognitive outcomes along with brain miRNA profiles. Finally, we tested if SI in aged females leads to changes in hippocampal cell proliferation.

## 2. Results

### 2.1. Post-Stroke SI Significantly Increased Mortality after MCAO

**Figure 1:**
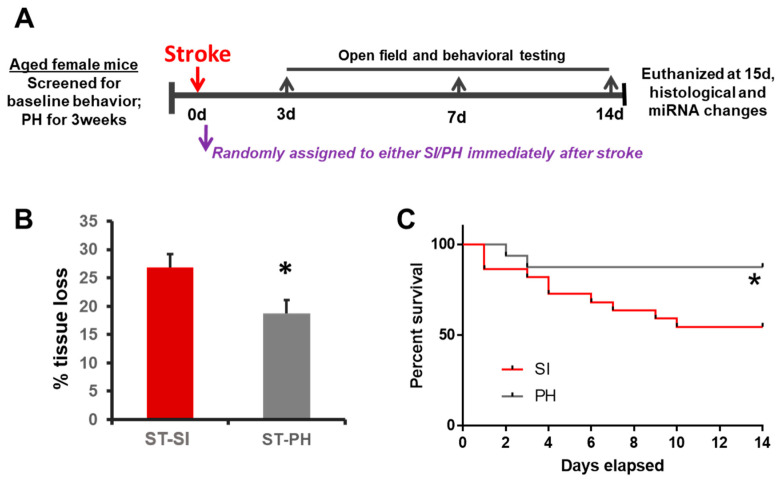
Social isolation immediately after stroke increases infarct size and mortality in aged female mice. (**A**) The experimental timeline illustrates the study design. Animals were screened for baseline behavior and immediately assigned to either pair-housing or isolation. The animals were randomly assigned and housed either in continued pair-housing or separated and housed singly (SI). Behavioral testing was performed on days 3, 7 and 14. Mice were euthanized on day 15 for infarct analysis. (**B**) Post-stroke SI (ST-SI) mice had significantly more atrophy compared to post-stroke pair-housed mice (ST-PH) (*n* = 9/group; * *p* = 0.024). Student’s t-test was used after the normality of data was confirmed. (**C**) The socially isolated mice had significantly higher mortality compared to pair-housed mice (*n* = 22 in SI; 16 PH initially. * *p* = 0.043). *P* value was obtained from the log-rank test.

In order to investigate whether post-stroke SI could influence ischemic injury size; mice were subjected to a 60 min MCAO and reperfusion. Mice were either isolated or remained paired for 15 days immediately after stroke (Figure 1A). At 15 days post-stroke, animals were euthanized, and brain tissue loss was evaluated with cresyl violet (CV) staining (Figure 1B). Infarct analysis revealed that immediately following a stroke, SI leads to a significant increase in tissue loss (26.87 ± 2.30% in SI vs. 18.75 ± 2.29% in PH; *n* = 9/group, *p* = 0.024). Significantly higher mortality was seen in the SI group compared to the PH group (45% in SI vs. 14% in PH; *p* = 0.043, Log-rank test) after a stroke (*n* = 22 in SI and 16 in PH at the start of the study) (Figure 1C).

### 2.2. SI Animals and Locomotor Activity after Stroke

**Figure 2:**
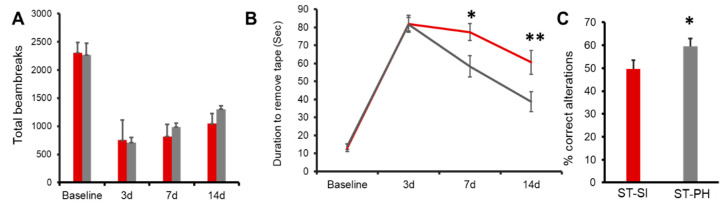
Post-stroke recovery is worse in isolated aged female mice compared to pair-housed mice. (**A**) Repeated open field analysis revealed that SI mice (red) had slower recovery compared to PH mice (grey). Although the SI mice showed no significant difference compared to PH mice, on day 14, the PH mice had a trend towards better locomotor activity compared to the SI mice (*n* = 12–13/group). (**B**) SI mice (red) also showed an increase in latency in the adhesive tape removal time compared to PH mice (grey) on day 14. (*n* = 12–13/group; * *p* = 0.020 at day 7, ** *p* = 0.005 at day 14 (**C**) The SI mice had a lower percentage of complete alteration in the Y-maze compared to the PH mice). ST-PH mice had significantly more% correct alterations compared to ST-SI mice (*n* = 12–13/group; * *p* = 0.05). Data are presented as mean ± SEM. Repeated two-way ANOVA was used to account for within-subject correlation for the repeated measures (A and B). Student’s *t*-test was used for group comparisons (**C**).

There was no significant difference seen at baseline or at day 3 after a stroke between SI animals compared to PH animals in spontaneous locomotor activity. At 14 days, there was a trend towards improved locomotor activity, but this was not significant between SI and PH animals; total beam breaks of 1043.7 ± 50.5 in SI group (*n* = 13) versus 1357.3 ± 109.9 in PH group (*n* = 12); (*p* > 0.05, two-way ANOVA) (Figure 2A).

### 2.3. SI Animals Had Deficits in Sensory-Motor Function

Animals subjected to SI immediately after stroke were tested for the changes in sensory-motor function by measuring the latency to remove an adhesive sticker [30]. Repeated measures ANOVA revealed a significant effect of housing, at day 7; 77.31 ± 4.69 s in SI group (*n* = 13) vs. 58.33 ± 5.91 s in PH group (*n* = 12), *p* = 0.020. These deficits were also seen at day 14; SI 60.59 ± 6.58 s (*n* = 13), vs. PH 38.75 ± 5.50 s (*n* = 12), *p* = 0.0054. These results suggest that SI impaired sensory-motor function and that PH improved performance in this task (Figure 2B).

### 2.4. SI Mice Had Worse Cognitive Outcomes

We next investigated whether SI after stroke altered cognitive outcomes compared to PH animals using the Y-maze test. While the total number of entries remained similar, consistent with the lack of change in overall motor behavior (SI 31.00 ± 1.32, PH 32.75 ± 1.57) between groups, SI mice had significantly less correct alterations; where one alteration is equal to the animal entering each arm once consecutively [31]. Data are expressed as the percent alterations over a 15-min period (*n* = 12–13/group; *p* = 0.05; repeated two-way ANOVA), (Figure 2C).

### 2.5. miRNAome Analysis Identified Differentially Altered miRNA after SI

**Figure 3:**
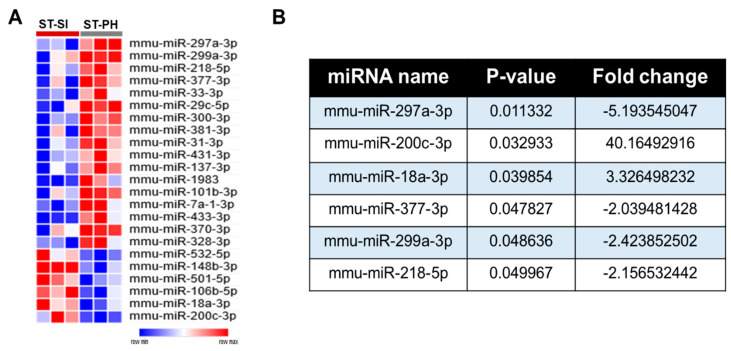
SI after stroke induces significant changes in brain miRNA profiles compared to PH. (**A**) 17 significantly upregulated and 6 downregulated miRNAs in the PH group compared to SI mice were identified and plotted as a heat map. (**B**) Further miRNAome analysis, based on the *p* value of *p* < 0.05 and a minimum fold change of 2 or higher, allowed us to identify the differentially regulated brain miRNAs between SI and PH stroke groups.

To examine the contribution of miRNAs to the detrimental effects of SI after stroke, we performed a whole miRNAome analysis in aged female mice after stroke. At post-stroke day 15, RNA was isolated from perilesional ipsilateral brain tissue for miRNAome analysis. We found several significantly altered brain miRNAs in SI compared to PH (Figure 3A). After setting a more stringent filter with a threshold of (>2-fold up or downregulation, and *p* < 0.05), we found 6 differentially expressed miRNAs between the two SI vs. PH stroke groups (Figure 3B), no significant changes in any of these miRNAs between sham groups. Comparative analysis for the effect of housing condition (pair-housed or isolated) determined the number of miRNAs affected by housing. Figure 3 shows the identified miRNAs.

### 2.6. Reduced Cell Proliferation in Post-Stroke SI Mice

**Figure 4:**
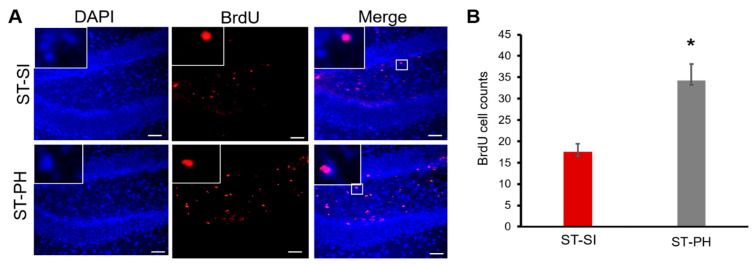
PH aged females had higher cell proliferation compared to SI animals after stroke. (**A**) Representative images of the hippocampus of brain sections from mice administered bromodeoxyuridine (BrdU) (day 3 to 7) after stroke. Immunohistochemistry analysis and co-labeling with anti-BrdU (in Red) and DAPI (in blue) showed that mice that were isolated immediately after a stroke had lower BrdU-positive cells in the dentate gyrus. Magnified images in insert show co-labeling of BrdU and DAPI. Scale bar denotes 75 µm (**B**) Quantification of these BrdU-positive cells showed a significant increase in cell proliferation in the dentate gyrus of ST-PH mice compared to the ST-SI mice (*n* = 4/group. * *p* = 0.008). Data are presented as mean ± SEM. Student’s t-test was used for group comparisons.

Our previous data support that SI after stroke can lead to a decrease in BDNF, a well-known neurotrophic factor [32]. Therefore, we examined cell proliferation, specifically in the ipsilateral dentate gyrus. At 15 days post-stroke, there was a significant reduction in the total number of bromodeoxyuridine (BrdU)-positive cells in the dentate gyrus of the hippocampal region in stroke females subjected to SI, suggesting reduced cell proliferation. Brain sections were assessed from coronal coordinates bregma ~−1.82 mm; −2.18 mm; −2.54 mm. Cell counts were quantified by an investigator blinded to housing conditions from the ipsilateral dentate gyrus of 3 brain sections per animal (*n* = 4/grp). Average cell counts revealed that SI animals had significantly fewer proliferating cells compared to PH mice (SI 17.5 ± 1.94, PH 34.25 ± 3.86; *n* = 4/grp), (* *p* = 0.008, Student’s t-test) (Figure 4B). Together, these findings suggest immediate post-stroke SI can reduce cell proliferation in dentate gyrus after stroke (Figure 4).

## 3. Discussion

There is a growing epidemic of lonely and socially disconnected people in our society. Meta-analyses have revealed that SI, loneliness and living alone has a significant effect on mortality that exceeds the risk associated with obesity [33] and other clinical risk factors [5]. Much of the burden of isolation falls on older women, in part due to their longer life expectancy. According to 2019 census data, 4.5 million men over the age of 65 live alone, compared to 9.4 million women. Older women are also at a higher risk for stroke, especially over the age of 75, again in part due to their longer life expectancy [34]. Our previous data have already shown that SI after stroke can lead to worse stroke outcome and increased mortality in male mice [14,35]. Not many in-depth studies have examined the detrimental effect of SI on stroke recovery in females [36].

This study demonstrates that immediate post-stroke isolation in aged females significantly worsens post-stroke behavioral recovery and increases mortality. Post-stroke SI leads to impaired functional recovery, as measured by deficits in the adhesive-tape removal test. In addition, SI mice showed significantly poorer cognitive outcomes in the Y maze. Although mice tested three days post-stroke showed no differences in locomotor activity, compared to PH mice, SI mice had significantly reduced cognitive function at post-stroke day 14, suggesting that continued SI is detrimental in a progressive manner following stroke. Impairment in neurogenesis appears to be an important underlying mechanism that was associated with impaired recovery in isolated mice [37,38]. Consistent with this idea, aged SI female mice also demonstrated significant changes in brain miRNA profiles involved in cell proliferation. Using in silico analysis, we found that SI alters neurotrophin and focal adhesion pathways, which may contribute to reduced hippocampal cell proliferation (Supplementary Figure S1). This suggests that SI worsens functional recovery in parallel to the changes in miRNAs and cell proliferation rates.

Prior studies have emphasized the importance of using non-pharmacological approaches to promote stroke recovery, one of which is enhanced social interactions [14,39,40]. In a prior study, we found that SI prior to the onset of stroke leads to significantly larger infarcts compared to mice that remained PH in young ovariectomized female mice [35]. However, these approaches are limited by the fact that most stroke patients are elderly and may become isolated after injury [32]. A stroke-induced disability could have long-lasting effects on health and mortality; therefore, it is important to determine if post-stroke SI influences chronic recovery in aged female animals to identify underlying mechanisms. In this study, we have found that similar to young female ovariectomized mice, aged and reproductively senescent female mice subjected to SI after stroke also have increased infarct size and higher mortality.

The higher amount of tissue loss in the SI mice could account for the delay in functional recovery. However, given that at 3 days post-stroke, both the SI and PH mice showed similar functional deficits supports the possibility that housing conditions have an effect on functional recovery independent of injury size, which grows in magnitude over time. Interventions to improve recovery in elderly stroke patients could thus target social factors, even after the immediate post-stroke period.

Cognitive impairment is common in both stroke survivors and in the elderly [41]. Consistent with these clinical findings, in a prior study, we found that SI, even in young mice, leads to poorer cognitive performance [42]. The Y-maze is specifically designed to measure the animal’s spatial learning and memory [31]. Animals with impaired cognition tend to revisit the same arm repeatedly or miss alterations compared to animals with normal cognition, and they typically explore a new arm rather than one previously visited [31]. The Y-maze test requires the animal to use the hippocampus, septum, basal forebrain and prefrontal cortex, all parts of the brain involved in processing memory and emotion [43]. This test is particularly valuable in assessing hippocampal function, crucial in learning and spatial memory [44]. When evaluated in the Y-maze at post-stroke day 14, SI mice had significantly less correct alterations than PH mice. This finding suggests that chronic isolation post-stroke can lead to an increase in cognitive impairment and worse post-stroke outcomes. These findings are consistent with previous studies suggesting SI may play an important role in cognitive disability. This could be a result of changes in neurotrophic factors such as BDNF and reduced neurogenesis [14]. BDNF is downregulated in both chronic isolation models and after stroke [45]. Studies examining changes in hippocampal BDNF levels and neurogenesis during stressful environmental exposure, such as chronic SI, found that miRNAs that target BDNF could be potential therapeutic targets for the treatment of anxiety and depressive-like behaviors that are often seen in patients after stroke [46,47,48]. To test this, we used miRNAome analysis to further examine changes in miRNA expression in aged, isolated animals after stroke.

We identified changes in miRNA expression profiles between SI and PH mice with stroke. Our findings show increases in miR-18a-3p and miR-200c-3p and decreases in miR-297a-3p, miR-299a-3p and miR-218-5p with SI compared to PH. Interestingly some of these miRNAs have been linked to cell proliferation, differentiation and inflammation in other disease models [49,50,51]. miR-200c-3p, while recognized as a regulator of cell proliferation and differentiation, with its overexpression growth-suppression, is seen [52]. Previous studies in cancer models have reported that upregulated miR-200c-3p inhibits expression of SOX2 to suppress proliferation and migration in renal cell carcinoma [53], and miR-200c-3p has also been shown to suppress proliferative and migratory capacities of nephroblastoma cells by targeting fibroblast growth factor receptor substrate 2 [54]. miR-18a is highly conserved in physiological processes such as proliferation, the cell cycle, apoptosis and differentiation, and has played a dual function in both promoting and inhibiting cancer progression [55]. Other studies have also shown epithelial miR-218-5p expression is negatively correlated to inflammation [56]. However, the significance of upregulation or downregulation of miRNAs following SI could have different effects in this model, leading to a decrease in overall cell proliferation.

Significantly altered miRNAs were used for the pathway analysis using the miRSystem, followed by KEGG database analysis, and predicted these miRNAs as potential regulators of cell proliferation and survival. The miRNAs identified to have altered expression in SI mice could be contributing to the differences in observed cell proliferation. Therefore, we directly examined changes in cell proliferation in SI and PH mice. We specifically looked at the hippocampal region due to its important role in cognitive function and given the deficits we observed on behavioral testing. Our findings of a marked decrease in cell proliferation in the hippocampus of SI mice is consistent with previous reports that show that chronic stress induced by SI causes a decrease in nerve growth factor (NGF) and BDNF, both of which are also important for neurogenesis, neural plasticity and post-stroke repair [57]. Decreases in post-stroke neurogenesis have been linked to poor functional outcome and mortality [58]. Given our results, the delayed recovery and impaired cognition seen in SI mice could be due to the reduced cell proliferation, exacerbated by the chronic stress of SI. As the number of people living alone is rising dramatically [8,9], identifying the contribution of miRNAs and the mechanisms involved in the detrimental effects induced by isolation will allow us to develop new approaches and therapeutic interventions to enhance functional recovery in elderly stroke survivors. Despite our interesting findings, this study does have a few limitations. It should be noted that the miRNA data were only collected at one time point, and we only had four BrdU animals at the 15-day time point and that our results remain correlative at this point. We also only analyzed mice subjected to stroke for behavioral outcomes. As sham mice do not have significant changes in behavior, and the primary comparison in this study was between pair-housed and isolated mice after stroke, not between stroke and sham, sham mice were not included in longitudinal behavior testing. The chronic assessment of behavior in stroke mice is essential to dissect out the SI-specific effect on recovery and is the most important for translation, as ultimately functional recovery is what matters most to patients. Future studies should include the use of antagomiRs (miRNA inhibitors) and mimics (miRNA enhancers) to understand the biological functions of the identified miRNAs in relation to neurogenesis.

## 4. Materials and Methods

### 4.1. Animals

Aged female C57BL/6 mice (16–18 months) were obtained from the National Institute on Aging (Bethesda, MD) and were acclimatized for at least 2 months in the animal care facility. Mice (18–20 months) were housed in pairs with a 12 h light/dark schedule in a temperature- and humidity-controlled vivarium, with ad libitum access to food and water. A total of 80 mice were pair-housed for a minimum of 3 weeks (2 mice/cage) with daily compatibility monitoring (e.g., loss of body weight, signs of fight wounds), 7 pairs of mice were excluded because of incompatibility. Mice were randomly assigned to SI or continued PH immediately following a stroke. Immediately after stroke surgery, we randomly assigned mice to either ST-PH (consisting of 1 stroke with 1 healthy partner mouse); or ST-SI (stroke isolated) [59]. The assigned housing conditions were maintained until the mice were euthanized. The Institutional Animal Care and Use Committee at the University of Texas Health Science Center at Houston approved all animal protocols, which we performed in accordance with the guidelines provided by the National Institutes of Health (NIH) and followed RIGOR guidelines.

### 4.2. Middle Cerebral Artery Occlusion

For focal transient cerebral ischemia, a midline ventral neck incision was performed under isoflurane anesthesia, and a 60-min unilateral right middle cerebral artery occlusion was performed by advancing a 6.0 silicone rubber-coated monofilament (Doccol Corporation, Sharon, MA, USA) from the external carotid artery stump to the root of MCA via internal carotid artery bifurcation [60]. Rectal temperatures were monitored, and body temperature is maintained at ~37 °C with an automatic heating system (Fine Science Tools, Foster City, CA, USA). Laser doppler flowmetry (DRT 4, Moor Instruments, Devon, United Kingdom) was used to measure cerebral blood flow to confirm occlusion (i.e., reduction of >80% compared to baseline reading). Following 60 min of occlusion, mice were re-anesthetized to remove the suture to restore blood flow. Sham mice were subjected to the same surgical procedure and exposure of the external carotid artery, but the suture was not inserted into the middle cerebral artery. The mice were euthanized at 15 days after stroke. All mice were given wet mash and 0.5 mL of saline once a day for at least 4 days following surgery to ensure adequate nutrition.

### 4.3. Sample Preparation and RNA Isolation and miRNAome Data Analysis

RNA extraction and qPCR were performed as detailed previously [59]. In brief, total RNA extracted from the perilesional ipsilateral cortex (from a separate cohort of mice, *n* = 12) using miRNeasy mini kits (Qiagen, Germantown, MD, USA) for miRNAome analysis or mirVana miRNA isolation kits (Thermo Fisher Scientific, Waltham, MA, USA) for other analyses, according to the suppliers’ protocols. RNA was stored at 80 °C until analysis by Exiqon (now part of Qiagen).

Amplification efficiency was calculated using the algorithms similar to LinReg software with Cq as the second derivative. We detected 388 differential miRNAs in all the samples; these were included in further analysis. The assays were required to be detected with 5 Cqs less than the negative control and with Cq < 37; for the Cq value of the global mean for each of the samples.

The mRNA targets of differentially expressed miRNAs were predicted using the publicly available miRSystem, a web-based system web tool [61]. Using embedded hyperlinks to the KEGG database, the top 3 identified pathways were used to predict cell proliferation as a potential mechanism.

### 4.4. Brain Atrophy Analysis

After 15 days following stroke, all animals were deeply anesthetized with an overdose of Avertin. Transcardial perfusion was performed using cold phosphate-buffered saline, followed by 4% paraformaldehyde. Brains were fixed overnight in PFA, transferred to 30% sucrose solution for cryoprotection for at least 24 h. Some brains were flash-frozen (*n* = 4–5/grp) for protein and RNA analysis, and these were not used for infarct quantification. Brains were cut into 30 μm sections using a microtome. Floating sections were stored at −20 °C until staining. For bromodeoxyuridine (BrdU) staining, a sub-cohort of mice (*n* = 4/grp) were injected with 75 mg/kg of BrdU (Sigma-Aldrich, St. Louis, MO, USA) once a day from days 3–7 after stroke to control for stress due to repeated handling and BrdU injections; all other mice received saline injections once a day. Eight sections at regular intervals from the appearance of the corpus callosum were mounted, stained with cresyl violet, and used for calculations of% total tissue loss compared to the contralateral hemisphere as described previously [62].

### 4.5. Open Field Analysis

Spontaneous locomotor activity was assessed using open field chambers. The mice were acclimatized to the testing room for 1 h prior to the test. Testing was performed during the light phase of the circadian cycle, between 9:00 am and 12:00 pm under normal fluorescent room lights. For testing, mice were individually placed in the open field chamber (15” × 15”) equipped with 16 infrared beam emitting LEDs on each side for a duration of 20 min. The total number of beam breaks was automatically collected by a computer-operated PAS open field system (San Diego Instruments, San Diego, CA, USA). The open field chambers were cleaned after each individual test session with 70% ethanol [63].

### 4.6. Adhesive-Tape Removal Test

The adhesive-tape removal test is a measure of somatosensory dysfunction after stroke in mice. For this, an adhesive-backed sticky tape (30 × 40 mm) was placed on the distal–radial region of the left wrist and the meantime to remove the tape was recorded [30]. Animals were trained for five days once a day prior to the stroke, and the latency to remove adhesive tape was measured on days 3, 7 and 14 after the stroke.

### 4.7. Y-Maze

To test the cognitive ability after the stroke, a Y-maze test was used. The Y-maze is specifically designed to measure spatial learning and memory. The mice were acclimatized to the room for 1 h. The mice were placed in the center of the maze. The order the arms were entered was recorded [31]. The Y-maze data were measured by the number of alterations completed in a 15-min trial, where one alteration is equal to the animal entering each arm once consecutively.

### 4.8. BrdU Labeling and Immunohistochemistry

Four mice from each group were injected with 75 mg kg/day of BrdU (bromodeoxyuridine/5-bromo-2’-deoxyuridine) once daily (intraperitoneal) on days 3 to 7 after stroke. The solution was prepared by dissolving BrdU (Sigma-Aldrich, St. Louis, MO, USA) in 0.007 N NaOH in 0.9% NaCl for injection. Mice were sacrificed and perfused on day 15; immunohistochemistry was used to identify and analyze BrdU-positive cells as in [62]. In brief, 30-micron brain coronal sections were obtained from each brain were slide mounted and incubated in blocking solution followed by microwave irradiation for 5 min in a 0.1 M, pH 6 citrate buffer solution. BrdU-positive cells were visualized by co-labeling with DAPI in Fluoromount. Sections were visualized under inverted light Zeiss Axiovert fluorescence microscope. Three images were taken from the striatum of coronal brain slices/animal (coordinates bregma ~−1.82 mm; −2.18 mm; −2.54 mm) (*n* = 4/group), were examined in each brain by a blinded investigator.

### 4.9. Statistical Analysis

Data expressed from individual experiments as mean ± SEM. We evaluated the data using Student’s t-test (for comparison between 2 experimental groups) or by repeated ANOVA (housing condition and day as variables) with post hoc correction for multiple comparisons. A log-rank test was used to compare the survival curves between groups. (GraphPad Prism Software Inc, San Diego, CA, USA). A *p* value of less than or equal to 0.05 was considered statistically significant. An investigator blinded to the experimental groups performed the data analyses.

## 5. Conclusions

In summary, post-stroke isolation leads to higher mortality, larger infarcts and delayed recovery in aged female mice. Analysis of brain tissue revealed significant changes in several miRNAs after SI compared to PH brains, specifically in miRNAs involved in cell plasticity and tissue repair. SI animals had significantly lower cell proliferation in the hippocampus compared to PH animals. Collectively, our data show that post stroke SI has a detrimental effect on stroke recovery.

## Data Availability

The data presented in this study are available from the first or corresponding author upon reasonable request.

## Supplementary Materials

The supplementary materials are available online at https://www.mdpi.com/1422-0067/22/1/99/s1.
