# Supplementary material for: Post-Stroke Social Isolation Reduces Cell Proliferation in the Dentate Gyrus and Alters miRNA Profiles in the Aged Female Mice Brain
Source: Int J Mol Sci. 2020 Dec 24;22(1):99. doi: 10.3390/ijms22010099 (PMC7795886; doi:10.3390/ijms22010099)
Supplement: Supplementary file 1 [file ijms-22-00099-s001.pdf]

Post-stroke social isolation reduces cell proliferation in the dentate gyrus and alters miRNA profiles in the aged female mice brain

Aleah Holmes, Yan Xu, Juneyoung Lee, Michael E Maniskas, Liang Zhu, Louise D. McCullough, Venugopal Reddy Venna

Supplemental Material

Supplemental figures (predictive pathway analysis)

Supplemental Figure 1A

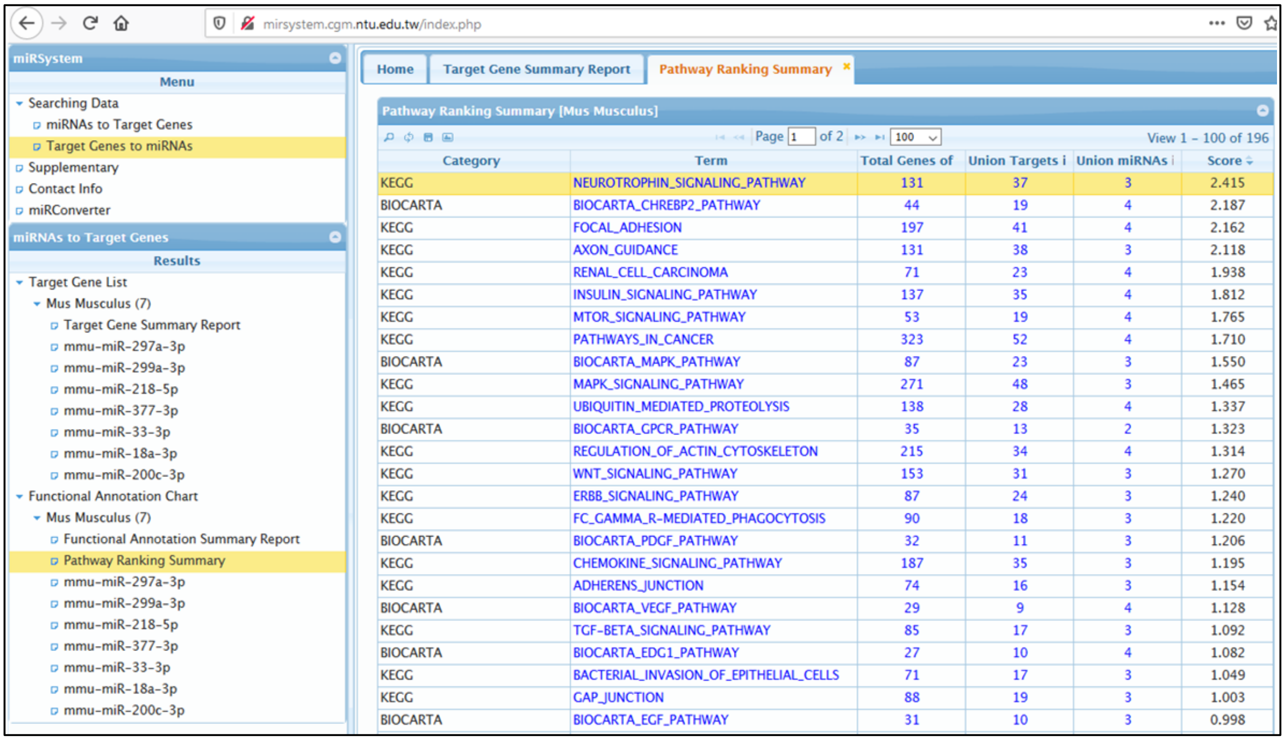

Category	Term	Total Genes of	Union Targets i	Union miRNAs	Score
KEGG	NEUROTROPHIN_SIGNALING_PATHWAY	131	37	3	2.415
BIOCARTA	BIOCARTA_CHREBP2_PATHWAY	44	19	4	2.187
KEGG	FOCAL_ADHESION	197	41	4	2.162
KEGG	AXON_GUIDANCE	131	38	3	2.118
KEGG	RENAL_CELL_CARCINOMA	71	23	4	1.938
KEGG	INSULIN_SIGNALING_PATHWAY	137	35	4	1.812
KEGG	MTOR_SIGNALING_PATHWAY	53	19	4	1.765
KEGG	PATHWAYS_IN_CANCER	323	52	4	1.710
BIOCARTA	BIOCARTA_MAPK_PATHWAY	87	23	3	1.550
KEGG	MAPK_SIGNALING_PATHWAY	271	48	3	1.465
KEGG	UBIQUITIN_MEDIATED_PROTEOLYSIS	138	28	4	1.337
BIOCARTA	BIOCARTA_GPCR_PATHWAY	35	13	2	1.323
KEGG	REGULATION_OF_ACTIN_CYTOSKELETON	215	34	4	1.314
KEGG	WNT_SIGNALING_PATHWAY	153	31	3	1.270
KEGG	ERBB_SIGNALING_PATHWAY	87	24	3	1.240
KEGG	FC_GAMMA_R-MEDIATED_PHAGOCYTOSIS	90	18	3	1.220
BIOCARTA	BIOCARTA_PDGF_PATHWAY	32	11	3	1.206
KEGG	CHEMOKINE_SIGNALING_PATHWAY	187	35	3	1.195
KEGG	ADHERENS_JUNCTION	74	16	3	1.154
BIOCARTA	BIOCARTA_VEGF_PATHWAY	29	9	4	1.128
KEGG	TGF-BETA_SIGNALING_PATHWAY	85	17	3	1.092
BIOCARTA	BIOCARTA_EDG1_PATHWAY	27	10	4	1.082
KEGG	BACTERIAL_INVASION_OF_EPITHELIAL_CELLS	71	17	3	1.049
KEGG	GAP_JUNCTION	88	19	3	1.003
BIOCARTA	BIOCARTA_EGF_PATHWAY	31	10	3	0.998

Supplemental Figure 1B

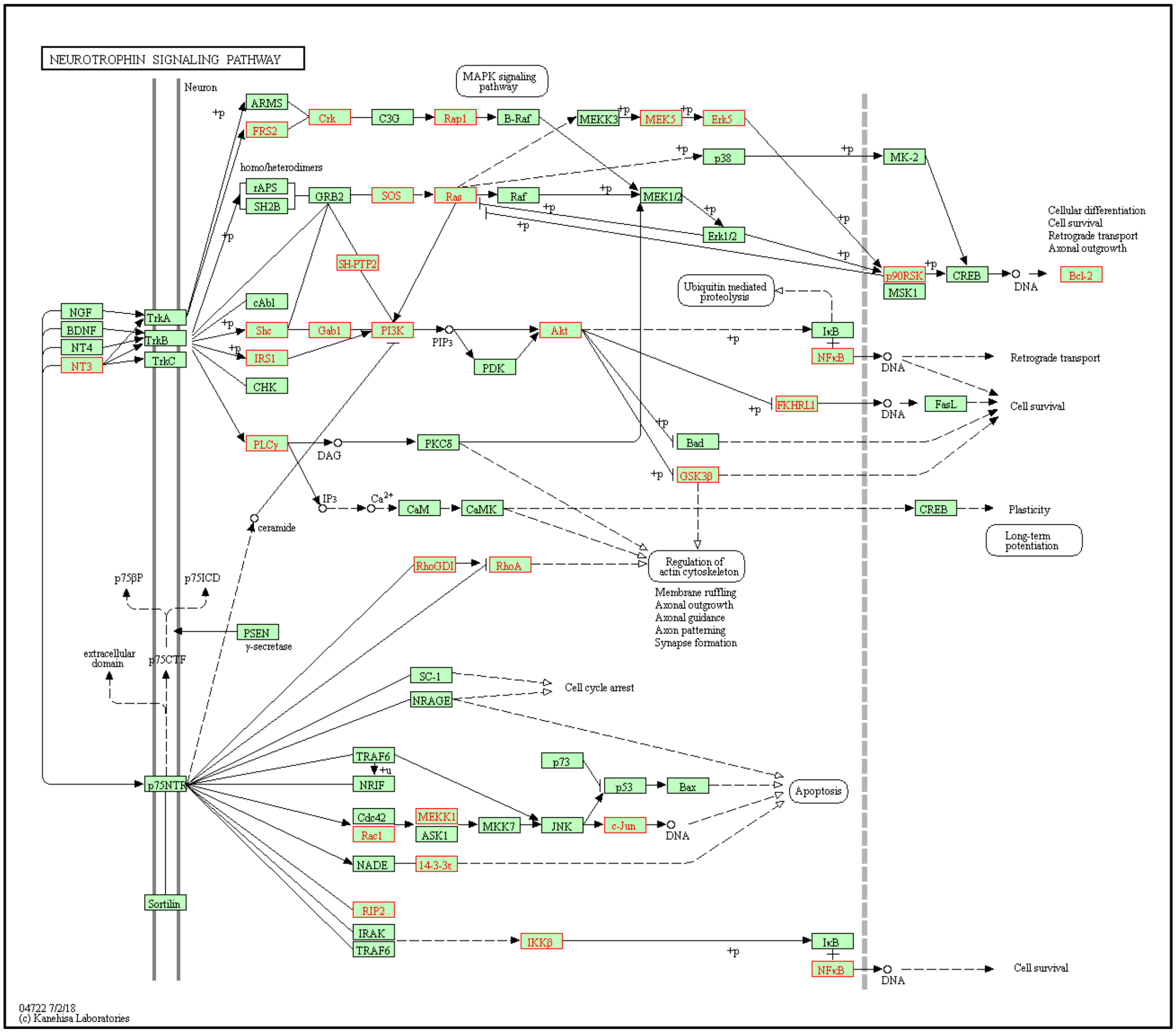

Supplemental Figure 1C

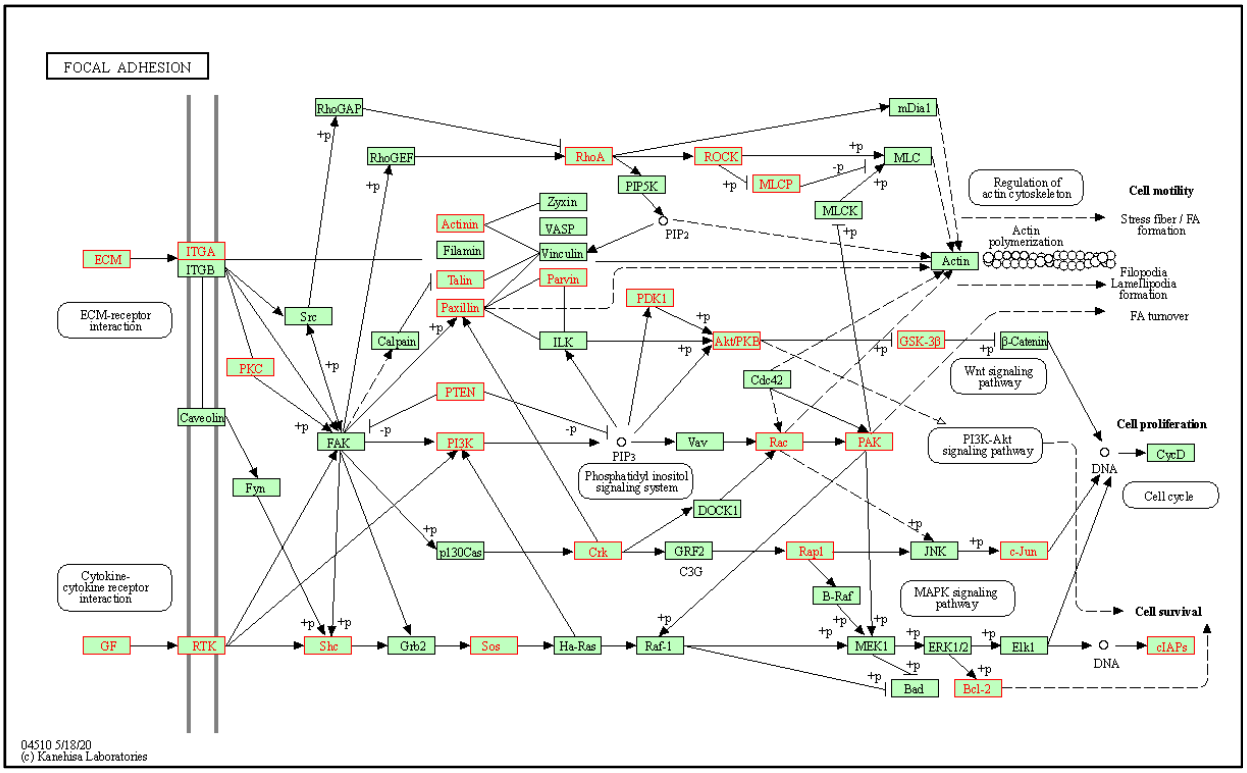

Supplemental Figure 1: The top significantly altered miRNAs were used for pathway analysis using the miRSystem, with KEGG and BIOCARTA pathway databases and preset parameters (Hit with ≥3; O/E ratio ≥2; Total genes in the pathway ≥ 25). Pathway rankings summary (**Fig. A**) revealed, that the Neurotrophin signaling pathway (**Fig. B**) and the Focal adhesion pathway (**Fig. C**) scored high (2.415 and 2.162 respectively). These pathways are involved in regulation of cell proliferation and survival.
